# Evolutionary history and genetic connectivity across highly fragmented populations of an endangered daisy

**DOI:** 10.1038/s41437-021-00413-0

**Published:** 2021-02-19

**Authors:** Yael S. Rodger, Alexandra Pavlova, Steve Sinclair, Melinda Pickup, Paul Sunnucks

**Affiliations:** 1 School of Biological Sciences, Clayton Campus, Monash University, Clayton, VIC Australia; 2 Arthur Rylah Institute, Department of Environment, Land Water and Planning, Heidelberg, VIC Australia; 3 Greening Australia Ltd, Perth, WA Australia

**Keywords:** Ecological genetics, Population genetics

## Abstract

Conservation management can be aided by knowledge of genetic diversity and evolutionary history, so that ecological and evolutionary processes can be preserved. The Button Wrinklewort daisy (*Rutidosis leptorrhynchoides*) was a common component of grassy ecosystems in south-eastern Australia. It is now endangered due to extensive habitat loss and the impacts of livestock grazing, and is currently restricted to a few small populations in two regions >500 km apart, one in Victoria, the other in the Australian Capital Territory and nearby New South Wales (ACT/NSW). Using a genome-wide SNP dataset, we assessed patterns of genetic structure and genetic differentiation of 12 natural diploid populations. We estimated intrapopulation genetic diversity to scope sources for genetic management. Bayesian clustering and principal coordinate analyses showed strong population genetic differentiation between the two regions, and substantial substructure within ACT/NSW. A coalescent tree-building approach implemented in SNAPP indicated evolutionary divergence between the two distant regions. Among the populations screened, the last two known remaining Victorian populations had the highest genetic diversity, despite having among the lowest recent census sizes. A maximum likelihood population tree method implemented in TreeMix suggested little or no recent gene flow except potentially between very close neighbours. Populations that were more genetically distinctive had lower genetic diversity, suggesting that drift in isolation is likely driving population differentiation though loss of diversity, hence re-establishing gene flow among them is desirable. These results provide background knowledge for evidence-based conservation and support genetic rescue within and between regions to elevate genetic diversity and alleviate inbreeding.

## Introduction

Biodiversity is declining globally at an unprecedented rate with tens of thousands of species facing impending extinction (Pimm et al., 2015; IUCN, 2020). Genetic diversity is an essential part of biodiversity, supporting populations’ persistence by promoting fitness and ability to adapt evolutionarily (Lande & Shannon, 1996). Thus, maintaining genetic diversity and evolutionary processes, such as gene flow and adaptive change, is critical for biodiversity conservation (Moritz, 1999; Crandall et al., 2000). Decreased gene flow under habitat fragmentation reduces the spread of novel genetic variants, lowering adaptive potential (Frankham et al., 2017). Genetic drift in small populations results in loss of genetic diversity and reduced efficiency of natural selection (Ellstrand & Elam, 1993; Frankham et al., 1999), leading to inbreeding depression and lowered adaptive potential, which elevate extinction risk (Frankham, 2005; Frankham et al., 2014). Thus, conservation management must consider conserving and augmenting genetic diversity and gene flow (Weeks et al., 2011; Frankham et al., 2017; Ralls et al., 2018).

Understanding the evolutionary processes underpinning a species’ distribution and population structure is essential for developing appropriate species-wide genetic management (Weeks et al., 2011; Pavlova et al., 2014). Both long-term divergence and short-term drift in recently isolated populations may result in population genetic structure. If populations are managed separately based on the incorrect assumption of genetic uniqueness rather than recent differentiation by genetic drift, extinction risk may increase by perpetuating the loss of genetic diversity through isolation and further drift (Coleman et al., 2013). Conversely, mixing populations that are too divergent can result in outbreeding depression, although this is infrequently observed, and its occurrence can usually be avoided by attention to risk factors including fixed chromosomal differences, adaptation to different environments and length of time since last gene flow (Frankham et al., 2011; Frankham 2015). Properly conducted genetic rescue is becoming widely accepted as a biodiversity conservation approach, particularly when the relative risks and benefits of mixing versus not mixing gene pools are properly assessed (Whiteley et al., 2015; Frankham et al., 2017; Ralls et al., 2018; Liddell et al., 2020). Accordingly, identifying the major processes underlying differentiation is important for informing risk-assessment frameworks and decision-support tools for maximising population persistence (Weeks et al., 2016). While these tools are increasingly available, and the data required more tractable to obtain, there remains much unfulfilled potential for evidence-based conservation decision making that embraces the importance of maintaining evolutionary processes (Liddell et al., 2020).

Understanding how best to reconnect fragmented populations requires an understanding of the levels of historical connectivity, which can be assessed using population genetic data (Mijangos et al., 2015; Breed et al., 2019). Research on genetic structure and past and present gene flow has been used to encourage admixture between populations of conservation concern, define seed transfer zones for restoring native grasslands and identify sources of genetic material to maximise evolutionary potential and increase restoration success (Knapp & Rice, 1996; Diekmann et al., 2010; Lloyd et al., 2011; Pacioni et al., 2015; Rodger et al., 2018; Potter et al., 2020). Despite the potential for population genetics to guide effective management, understanding of past and present connectivity is missing for many species. Even when relevant data exist, it remains rare for management-relevant interpretations of population genetic data to be articulated in terms likely to be useful to managers (Liddell et al., 2020).

The Button Wrinklewort (*Rutidosis leptorrhynchoides*, also spelled *leptorhynchoides*) is a perennial plant in the daisy (Asteraceae) family, endemic to Australia, and that was once widespread in grassy ecosystems in south-eastern Australia (Scarlett & Parsons, 1990). Since European human colonisation of its habitat in the early 1800s, the species has undergone severe reductions in population size and number, and is currently listed as endangered under the national Environmental Protection and Biodiversity Conservation Act 1999. Its known original range covered three main areas, each with breaks in distribution of approximately 500 km: the Canberra region in Australian Capital Territory (ACT) and New South Wales (NSW), west of Melbourne in Victoria (VIC), and on the Gippsland Plains in the east of VIC where it is now extinct (Morgan, 1995; Young et al., 1999). Highly fragmented populations now persist in habitat protected from farming and urbanisation (Scarlett & Parsons, 1990). The current range of *R. leptorrhynchoides* comprises approximately 31 small disjunct natural populations, almost half of which contain <200 individuals, and none exceeding 100,000 (NSW OEH 2012 and subsequent unpublished census data from regional agencies). In an attempt to stem population decline, management actions have been undertaken including habitat restoration, monitoring and supplementary planting.

Previous research using nine allozyme loci (for 551 individuals) and ten microsatellites (for 364 individuals) showed differentiation among sampled populations of only *F*_ST_ = 0.17 for allozymes and 0.03–0.14 for microsatellites, despite large breaks in the species’ distribution. This was interpreted as suggesting a high degree of gene flow at least until recently (Young et al., 1999; Pickup et al., 2012). The lack of a major genetic break in neutral markers matching the large geographic disjunction is not unique in the literature for plant taxa with similar distributions in south-eastern Australia such as *Swainsona recta* and *Senecio macrocarpus* (Buza et al., 2000; Ahrens et al., 2015). However, lack of strong population genetic subdivision could represent historical (pre-fragmentation) rather than contemporary genetic connectivity (Young et al., 1999), so population genetic datasets with greater resolution are required to assess gene flow. Genetic management of *R. leptorrhynchoides* has been proposed as part of its recovery plan (National Recovery Plan 2012). Current recommendations are to prioritise conservation of large, genetically diverse populations, because these will likely be the best sources for genetic rescue of smaller, less genetically diverse populations from similar environments, as supported by extensive crossing experiments including in the wild (Young et al., 1999; Pickup & Young, 2008; Pickup et al., 2012, 2013). A detailed understanding of which populations are the most genetically diverse and which populations are most vulnerable is needed for targeted management. Furthermore, insights into the distribution of genetic variation and evolutionary processes such as past divergence and gene flow are necessary for defining populations for conservation prioritisation (Liddell et al., 2020).

In this study we use a dataset of 12,965 single nucleotide polymorphism (SNP) loci to investigate the evolutionary history of *R. leptorrhynchoides* and describe patterns of genome-wide diversity and structure in populations across its distribution. We aimed to: (1) quantify levels of genome-wide differentiation between sampling locations and explore the underlying processes driving this differentiation; (2) assess levels of genome-wide genetic diversity within sampling locations and test whether they accord with previously published allozyme and microsatellite-based estimates; (3) infer some key parameters of population genetic history: population tree topology, divergence times and effective population sizes (scaled by mutation rate) were estimated by a coalescent tree method based on SNPs, and the role of gene flow in population isolation or admixture was explored via a maximum likelihood population tree based on SNP allele frequencies; and (4) use these results to define populations for conservation management planning. The molecular approaches we apply are likely to be affordable in many management scenarios, can be outsourced, do not require existing genome resources and hence are applicable to a wide range of species.

## Materials and methods

### Study species

**Fig. 1:**
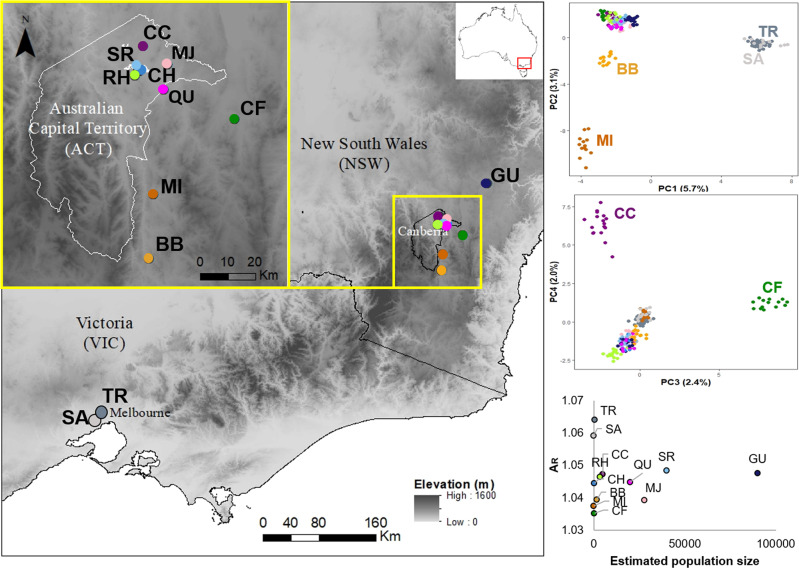
Distribution of sampling locations for *R. leptorrhynchoides*. Principal coordinate analysis (PCoA) derived from 12,965 SNPs for all 12 sampled populations. Population codes correspond to those presented in Table 1.

*Rutidosis leptorrhynchoides* is a perennial herb endemic to south-eastern Australia. It occurs in grassy eucalypt woodlands and treeless grasslands, most of which are dominated by kangaroo-grass, *Themeda triandra* (Morgan, 1995). The species has a lifespan of greater than 10 years in the field and is pollinated primarily by insects of modest flight distance such as *Lasioglossum* bees (Morgan, 1995; Courtice et al., 2020). Seeds are weakly wind-dispersed, most falling close to the parent plant (<50 cm) and persisting for only a short time in the soil seed-bank (Morgan, 1995). The lack of regular long-distance robust dispersal is evidenced by the fine-scale spatial genetic structure (Wells & Young, 2002). *Rutidosis leptorrhynchoides* has a single-locus sporophytic self-incompatibility system, in which pollen is identified as incompatible by the stigma and rejected when the pollen parent genotype at the *S*-locus carries alleles that match those at the *S*-locus receiving plant (Young et al., 2000; Mable et al., 2005). Reduction in *S*-allele diversity is detrimental for population fitness (Young & Pickup, 2010). The species exhibits variation in chromosome number across its distribution, with northern and easternmost southern populations being diploid (2*n* = 22) and majority of southern populations being autotetraploid, often with additional non-diploid karyotypes (Young et al., 1999). Here we focus solely on diploid populations, of which only two natural ones are known to remain in VIC in southern Australia, and ~500 km to the north there are 11 in ACT and 10 in NSW (Fig. 1). The tetraploid/non-diploid populations, not considered here, occur in a coherent geographic range in VIC, >70 km west from the last two known diploid populations in the region. Mixed-ploidy crosses are possible but rare in nature, resulting in offspring with triploid and aberrant chromosome numbers, which are predominantly sterile (Brown & Young, 2000; Young & Murray, 2000; Murray & Young, 2001). We therefore treat diploid populations as a discrete genetic management unit, separate from polyploid populations.

### Sampling, genotyping and SNP data filtering

Leaf material was collected from a total of 199 individuals from 12 locations (10 ACT/NSW, 2 VIC) across the current distribution of diploid *R. leptorrhynchoides* in 2017/2018 (Fig. 1 and Table 1). Where practical, we sampled every population using a standard layout, defined by a rectangle measuring 20 × 15 m, containing 20 sample points in three rows, spaced at 5 m intervals: a row at 0, 5, 10, 15, 20 m on the long axis, repeated at 0, 5, 10, 15 m on the short axis. We sampled genetic material from the plant closest to each point, avoiding new recruits judged <1 year old. Some sampling locations (BB, CF, CH, MI, SA) had plants over too small an area to accommodate this grid, so we sampled from across the population, as close to the 5 m grid arrangement as we could. Two populations (MI, SA) were so small numerically (13 and 18 individuals, respectively, Table 1) that we sampled from every plant we could locate on the day of sampling. One population (CH) was widely dispersed in many small clusters, and to maximize consistency with the other samples, we sampled all plants from the largest cluster. For one location (TR) that is a particular focus for managers, in addition to the standard layout, we collected samples from plants around the population periphery.

**Table 1:** List of sampling locations, state/territory, sample size (*N*), estimated population size from the National Recovery Plan 2012.

Location	Code	State/territory	*N*	Latitude	Longitude	Census size
Gundary	GU	NSW	20	−34.8	149.74	~90,000
Crace	CC	ACT	17	−35.23	149.13	~5000
Majura	MJ	ACT	17	−35.29	149.21	~28,000
Stirling Ridge	SR	ACT	17	−35.3	149.12	~40,000
Capital Hill	CH	ACT	6	−35.31	149.12	293
Red Hill	RH	ACT	15	−5.32	149.11	~3440
Queanbeyan	QU	ACT	15	−35.37	149.2	~20,000
Captains Flat	CF	NSW	14	−35.46	149.43	306
Michelago	MI	NSW	13	−35.7	149.17	13
Bredbo	BB	NSW	14	−35.91	149.15	1694
Truganina	TR	VIC	39	−37.83	144.72	591^a^
St Albans	SA	VIC	12	−37.74	144.8	18^a^
Total			199			

Locations are shown in Fig. 1.

^a^Recent census counts.

Leaf samples were dried and sent to Diversity Arrays Technology Pty Ltd for DNA extraction and genotyping using the DArTseq^TM^ (reduced-genome representation) platform (Jaccoud et al., 2001). DArTseq^TM^ is similar to double-digest restriction-associated sequencing with the workflow optimised to lower rates of missing data, and a quarter of samples being re-analysed starting from library preparation step. Each locus is given a repeatability score based on sequencing of different libraries for the same samples, providing a basis for selecting markers with very low error rates (Georges et al., 2018). More details of DArTseq^TM^ can be found in Supplementary Methods S1.

The raw genomic dataset comprised 53,758 codominant, genome-wide, biallelic SNPs. Data filtering was done using the DARTR package (Gruber et al., 2018) in *R* v3.5.0 (R Core Team, 2018). We removed 25,288 loci that were not 100% reproducible. With this selection criterion, nearly all low-frequency alleles should be reliable. In addition, we removed 11,779 loci with >25% missing data per locus and 51 *F*_ST_ outliers (see below), and 4 individuals with >15% missing data (along with 35 monomorphic loci associated with them). To control for very close physical linkage, we retained only one SNP per sequenced fragment (~69 bp), which removed a further 3640 loci. We also filtered for monomorphic loci, but found none.

To identify significant *F*_ST_ outliers, which could have evolved under selection and are likely to violate a Wright–Fisher model assumed in many approaches used, we ran BAYESCAN v. 2.1 (Foll & Gaggiotti, 2008). The analysis was performed with 20 pilot runs each consisting of 5000 iterations, followed by 100,000 iterations with a 50,000 burn-in and a sample size of 5000. Prior odds for the neutral model were set to 100, and the *F*_IS_ prior was set to ‘uniform between 0.0 and 0.4’ based on the range of typical values for this inbreeding statistic for *R. leptorrhynchoides* (Pickup et al., 2012). The false discovery rate threshold for outlier locus detection was set at 0.05. We found 51 significant outliers that were removed from the dataset.

The final filtered SNP dataset used for analyses contained 12,965 high-quality biallelic SNP loci with only 7.62% missing data. A second dataset of 1889 SNP loci with no missing data was also created for use in some analyses (below).

### Analysis of population genetic differentiation

We inferred population structure by estimating the likely number of genetic clusters (*K*) and membership of each individual in each genetic cluster using STRUCTURE v2.3.4 (Pritchard et al., 2000). To preclude inferences influenced by missing data, STRUCTURE was run using the 1889 SNP dataset with no missing data. Ten independent replicate runs were performed for each of *K* = 1–12 genetic clusters (which would have been increased if warranted by the higher of these *K* estimates), each with a burn-in of 500,000 followed by 1,000,000 Markov chain Monte Carlo (MCMC) iterations. We used the admixture model without prior population information. To identify further patterns of substructure, we performed additional STRUCTURE runs on only northern (ACT/NSW) populations, which belonged to a single cluster based on *K* = 2 analysis, testing a range of genetic clusters from *K* = 1 to 10 (Janes et al., 2017). CLUMPAK software (Kopelman et al., 2015) was used to summarise and visualise STRUCTURE output and perform the Evanno Delta *K* method (Evanno et al., 2005) and ln Pr(*X*|*K*) method of Pritchard & Wen (2003) of determining best *K*. Based on the recommendations of Janes et al. (2017), we assessed the population structure and number of genetic clusters by applying and comparing these two methods. We checked that conclusions did not differ materially when using the full dataset in fastSTRUCTURE (Raj et al., 2014), and they did not (justification in Supplementary Figs. S11–S14).

To quantify genetic differentiation among populations, we estimated pairwise Weir & Cockerham (1984) *F*_ST_ in the *R* package HIERFSTAT (Goudet, 2005) using the full 12,965 loci dataset. In addition, a hierarchical analysis of molecular variance (AMOVA) was conducted in *R* using the ‘poppr.amova’ function of POPPR (Kamvar et al., 2015), with significance testing using 999 permutations. Populations were grouped into geographic regions: VIC (consisting of TR and SA) and ACT/NSW (consisting of BB, CC, CH, CF, GU, MI, MJ, QU, RH and SR). Principal coordinate analysis (PCoA) was performed in the *R* package DARTR (Gruber et al., 2018). PCoA analysis was also performed on the no-missing dataset and yielded similar results (Supplementary Fig. S15). We tested for an isolation by distance model of differentiation by plotting geographic distance versus *F*_ST_ / (1 *–* *F*_ST_) and performing a Mantel test using the function ‘gl.ibd’ in DARTR for all 12 populations and for populations only within the ACT/NSW region.

### Estimation of within-population diversity

Observed heterozygosity (*H*_o_), gene diversity (*H*_S_), allelic richness (*A*_*R*_) and *F*_IS_ for each population were estimated using HIERFSTAT. Genetic diversity analyses were also performed on the no-missing dataset and yielded similar results (Supplementary Fig. S16). To gain some insight into how different the VIC and ACT/NSW populations are genetically, we estimated the frequency of private alleles per region using the R package POPPR (Kamvar et al., 2015). Individuals were grouped into their respective regions (VIC or ACT/NSW) and the frequency distributions of alleles unique to each region were assessed to determine whether the majority of unique alleles are rare or common within each sample.

A correlation between low diversity and high population-specific *F*_ST_ indicates that loss of diversity through genetic drift drives apparent differentiation (Coleman et al., 2013). To test for evidence of drift in our data, we calculated mean population-specific *F*_ST_ for each population in GESTE v. 2.0 (Foll & Gaggiotti, 2006) and performed a simple linear regression against genetic diversity (*A*_*R*_ and *H*_o_).

### Population history and connectivity

To explore history of population divergence, we inferred a population tree using a coalescent model and Bayesian MCMC approach implemented in SNAPP (Bryant et al., 2012) run using BEAST 2.6 (Bouckaert et al., 2019). SNAPP’s model builds coalescent trees for each SNP locus independently and then integrates over all possible genealogies to provide estimates of tree topology and parameters of population divergence times and effective population sizes, scaled by mutation rate. The SNAPP model assumes divergence without gene flow among genetic populations. For SNAPP analyses to complete in feasible computational times, we used reduced SNP datasets of 2000 loci randomly chosen from the full 12,965 loci dataset and four randomly selected individuals to represent each population (Spalink et al., 2019; Rojas et al., 2020). We used the default prior and model parameters (*µ* = 1, *v* = 1, coalescence rate = 10, priors: *α* = 11.75, *β* = 109.73, *κ* = 1, *λ* = 0.00765) and ran two independent replicate MCMC runs of 1,000,000 iterations with sampling every 1000 steps and a burn-in of 10%. To ensure the choice of individuals and loci did not influence our results, this analysis was repeated on another set of randomly sampled individuals and 2000 randomly sampled loci.

We used TRACER v. 1.7.1 (Rambaut et al., 2018) to visually inspect the output for acceptable mixing and replicate run convergence, confirming effective sample sizes >200 for all parameters. The two runs for each replicate were combined using LOGCOMBINER (Drummond & Rambaut, 2007), using a burn-in of 10%. The posterior distribution of gene (SNP) trees was visualised using DENSITREE v. 2.6.1 (Bouckaert, 2010) and summarised by generating a maximum clade credibility (MCC) tree using TREEANNOTATOR v. 2.6 (Drummond & Rambaut, 2007).

To test whether historical or contemporary migration resulting in gene flow could explain variation in addition to that explained by populations diverging in isolation, and to reveal potential admixture events, we used TreeMix v1.13 (Pickrell & Pritchard, 2012) on the full 12,965 SNP dataset. TreeMix uses allele frequency data to approximate an unrooted maximum likelihood population tree. A stepwise likelihood procedure is used to test the effect of migration on the residual covariance matrix and determine the optimal placement of migration events in the population tree. We inferred a topology without admixture, as well as allowing up to five migration events.

## Results

### Levels of differentiation and diversity in *R. leptorrhynchoides*

Pronounced genetic differentiation between VIC versus ACT/NSW sampling locations for *R. leptorrhynchoides* was supported by several different analyses. The first axis (PC1) of the PCoA analysis explained 6.17% of the total variance and clearly separated VIC locations from ACT/NSW (Fig. 1). Additional axes, PC2, PC3 and PC4, separated out ACT/NSW sampling locations, accounting for 3.05%, 2.4% and 1.9% of the total variation, respectively. The NSW locations BB and MI were the next most differentiated from the rest of the northern ones according to PC2 (Fig. 1). No clear differentiation was observed between the two VIC locations across the first six PC axes (Supplementary Fig. S1).

**Fig. 2:**
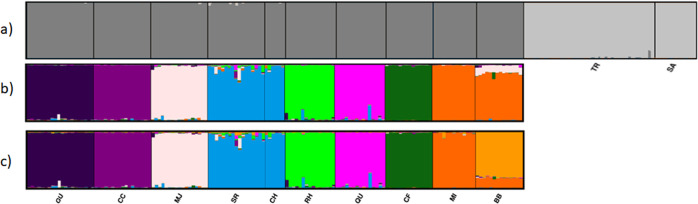
STRUCTURE clustering of *R. leptorrhynchoides* individuals based on 1889 SNPS. Plots for **a** all 12 populations with an optimum *K* value of 2, **b** ACT/NSW populations only (which formed part of a single genetic cluster when *K* = 2 for all 12 populations) for *K* = 8, and **c** *K* = 9. Each vertical bar corresponds to an individual and the vertical axis is membership (*Q* value) in each of the *K* genetic clusters. Population codes correspond to those presented in Table 1.

In the STRUCTURE analysis on all 12 sampling locations, the optimal value of *K* was 2 according to the Delta *K* method, which usually reflects the top level of structure in the data (Janes et al., 2017) (Supplementary Fig. S2), with VIC forming one cluster and ACT/NSW a second. Although output for *K* > 2 consistently separated southern (VIC) and northern (ACT/NSW) locations, the runs were insufficiently consistent to determine a best *K* according to ln Pr(*X*|*K*) method (Supplementary Fig. S3). In the analysis of ACT/NSW alone, the Delta *K* method supported the presence of nine genetic clusters, and the ln Pr(*X*|*K*) method supported eight (Supplementary Fig. S4). With *K* = 8–10, almost all sampling locations were assigned to their own cluster, with the exceptions that SR and CH, <1 km apart, were assigned to the same cluster (Fig. 2; for all ACT/NSW STRUCTURE outputs refer to Supplementary Fig. S5).

When sampling locations were grouped into geographic regions, AMOVA revealed that grouping sampling locations into the north and south regions explained 8.08% of variance (*ϕ*_CT_ = 0.081, *p* = 0.001), and sampling locations within regions explained 21.50% (*ϕ*_SC_ = 0.234, *p* = 0.001). Similarly, pairwise *F*_ST_ comparisons showed significant pairwise differentiation among most sampled locations, with largest values observed for comparisons involving MI and CF in NSW, and SA in VIC (Table 2). No significant isolation by distance was detected among all locations (*r* = 0.294, *p* = 0.109, Supplementary Fig. S6a) or among those in ACT/NSW alone (*r* = 0.481, *p* = 0.057, Supplementary Fig. S6b).

**Table 2:** Pairwise *F*_ST_ values among sampled populations of *R. leptorrhynchoides* based on 12,965 SNPs.

Population	GU	CC	MJ	SR	CH	RH	QU	CF	MI	BB	TR
CC	0.13										
MJ	0.1	0.13									
SR	0.08	0.11	0.080								
CH	0.11	0.14	0.13	0.05							
RH	0.11	**0.14**	0.12	0.08	0.11						
QU	0.110	**0.14**	0.11	**0.09**	**0.12**	**0.12**					
CF	**0.19**	**0.22**	**0.21**	**0.17**	**0.22**	**0.210**	0.2				
MI	**0.23**	**0.25**	**0.25**	**0.22**	**0.26**	**0.25**	**0.24**	**0.32**			
BB	**0.14**	**0.17**	**0.150**	**0.12**	**0.17**	**0.16**	0.15	**0.240**	**0.23**		
TR	**0.12**	**0.15**	**0.13**	**0.11**	**0.12**	**0.14**	**0.13**	**0.19**	0.22	0.16	
SA	**0.16**	**0.2**	**0.19**	**0.16**	**0.16**	**0.19**	**0.18**	**0.26**	**0.29**	**0.21**	**0.04**

Bold values are significant (*p* < 0.05). Population codes correspond to those presented in Table 1.

**Fig. 3:**
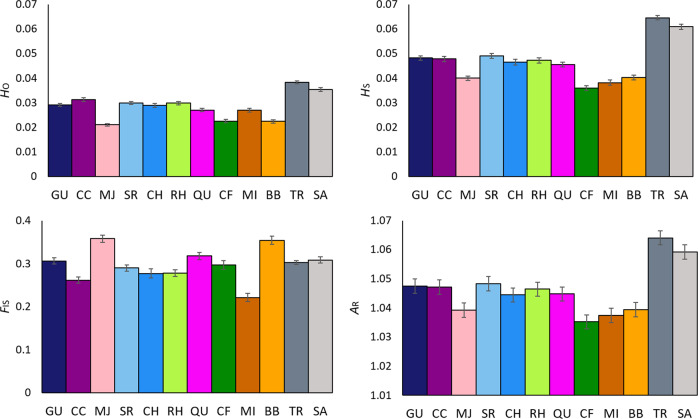
Summary of genetic diversity parameters for *R. leptorrhynchoides* populations sampled in this study, estimated from 12,965 loci. Population codes correspond to those presented in Table 1. *H*_o_ observed heterozygosity, *H*_S_ gene diversity, *A*_*R*_ allelic richness. Numerical values are presented in Supplementary Table S3.

Genetic diversity and population size estimates (Table 1) were not correlated (*r*^2^ = 0.007, *p* = 0.80, Fig. 1 and Supplementary Fig. S7). The VIC locations TR and SA had the highest heterozygosities (Fig. 3). In contrast, comparably small populations at the southern end of the northern range—BB, CF and MI—had the lowest levels of genetic diversity by all measures. It is also notable that MJ had one of the lowest *H*_O_ (0.021) and relatively low *A*_R_ (1.14) even though it is one of the larger populations, estimated to number >28,000 plants (Table 1, Fig. 1 and Supplementary Fig. S7).

The majority (62.0%) of the 25,930 alleles in the full dataset were shared in common between samples from ACT/NSW and those from VIC. While 19.8% of the 25,930 were present only in ACT/NSW, and 18.3% were unique to VIC, most of these private alleles were rare, representing <5% of the sample for a given marker (Supplementary Fig. S8). Some very rare alleles may not have been adequately detected with the sample size of 51 individuals in VIC but alleles >5% in the population should have been detected >95% of the time (Sjogren & Wyoni, 1994).

### Divergence between genetic populations is consistent with genetic drift in isolation

**Fig. 4:**
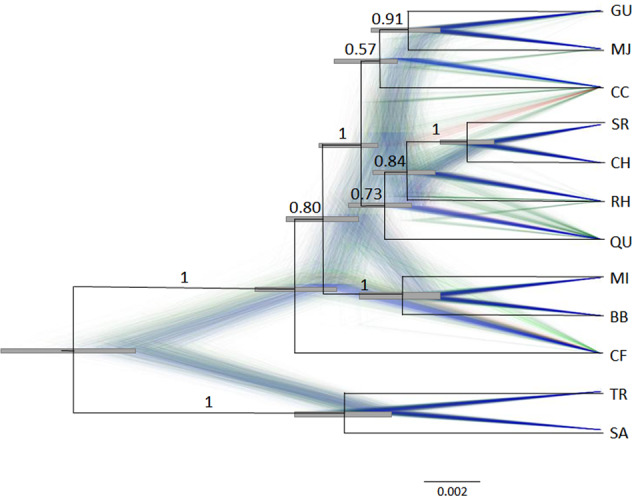
Combined SNAPP population tree of *R. leptorrhynchoides* populations based on 2000 SNPs. Maximum clade credibility tree generated in FigTree v. 1.4.4 (https://github.com/rambaut/figtree/releases) is in black with 95% HPD for height at each node indicated by the grey bar. Branch lengths are measured in expected substitutions per site. Densitree of superimposed gene trees recorded during the MCMC analysis visualises the range of alternative topologies, indicative of past gene flow. Gene trees shown in blue lines indicate most frequent trees, next most frequent are red, third most are green. Numbers represent posterior probabilities of branches. Population codes correspond to those presented in Table 1.

There was strong support for a genetic split between VIC (TR and SA) and all northern locations, according to the SNAPP MCC population trees produced by the two different datasets (posterior probability PP = 1 for VIC and ACT/NSW branches, Fig. 4 and Supplementary Fig. S9). All clades that showed PP > 0.75 were supported by both datasets. CF is shown as an ancestral population of the rest of ACT/NSW populations, which themselves form a well-supported clade (PP = 1). The grouping together of the two locations in the southern part of the species distribution in NSW (BB and MI) also received high support (PP = 1), as did the two sites in ACT that are near each other, SR and CH (PP = 1). Relationships between GU and the cluster of ACT populations (all of the latter are in close geographic proximity) are not well resolved, likely due to past gene flow. Estimates of average effective population size over time scaled by mutation rate (*θ*) were highly correlated with estimates of genetic diversity per population (Supplementary Table S3 and Supplementary Fig. S18).

**Fig. 5:**
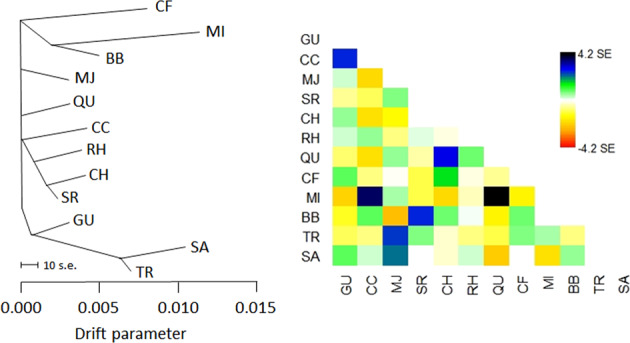
TreeMix analysis of *R. leptorrhynchoides* based on 12,965 SNPs showing the unrooted maximum likelihood tree, and the residual fit from the tree. Drift parameter is shown on the *x*-axis and the scale bar shows 10 times the average standard error of the entries in the sample covariance matrix. Large, positive residuals (blue-black colours) indicate population pairs that are more closely related to each other in the data than suggested by the best-fit tree, and may be candidates for admixture events.

**Fig. 6:**
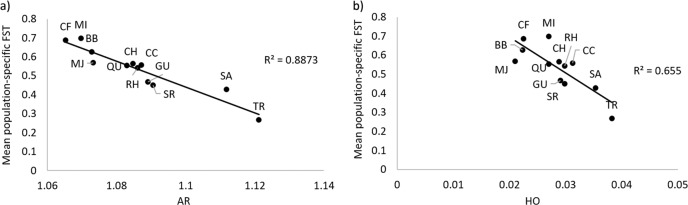
Regressions between mean population-specific *F*_ST_ and estimates of genetic diversity using 12,965 SNPs. **a** Allelic richness *A*R vs mean population-specific *F*_ST_ and **b** observed heterozygosity HO vs mean population-specific *F*_ST_.

The unrooted maximum likelihood tree inferred by TreeMix (with no migration events added) showed topology largely concordant with population clustering inferred by the PCoA, STRUCTURE and SNAPP analyses (Fig. 5). The two VIC locations are grouped together, divergent from ACT/NSW, and CF, MI and BB in NSW are grouped close together and show high levels of drift, as indicated by the length of horizontal branches, which are proportional to the amount of genetic drift that has occurred since a population became isolated. The inference of strong drift processes was supported by the population-specific *F*_ST_ analysis, as follows. Populations that were more genetically distinct had lower genetic diversity: regression of the mean population-specific *F*_ST_ against *H*_O_ and *A*_R_ yielded strong negative relationships (*R*^2^ = 0.655, *p* = 0.001, and *R*^2^ = 0.882, *p* < 0.001, respectively, Fig. 6), suggesting that drift drives the apparent differentiation in populations.

The strongly positive TreeMix residuals among some pairs of locations indicate that these may be more closely related than they appear in the tree, and are thus candidates for potential admixture events (Fig. 5). Tree topology did not change significantly with the addition of subsequent migration events, although the fit of the data was optimised with three migration edges (Supplementary Fig. S10). The low weights of these proposed migration events are related to the low proportion of alleles in the descendent populations that are derived from the ancestral population and may be reflective of past gene flow (Pickrell & Pritchard, 2012). Past gene flow between GU and CC is supported by their close grouping and the low support for their split in the SNAPP tree (PP = 0.57, Fig. 4).

## Discussion

### Major genetic split between northern and southern populations reflects geography

We found clear genetic differentiation between the southern (VIC) and northern (ACT/NSW) populations of *R. leptorrhynchoides*, which was hinted at but not resolved in previous population genetic studies of this species (Pickup et al., 2012, 2013). This probably reflects the relatively low resolution of the techniques previously available, and highlights the value of genome-wide SNPs for improving our understanding of fine population genetic structure. The observed genetic split reflects the break in the current geographic distribution of the species. This discontinuity in distribution is mirrored by many other southern Australian species native to grassy woodlands and grasslands dominated by tussock-forming grasses, including plants (Department of the Environment, 2020; Buza et al., 2000; Sinclair, 2010), insects (Clarke & O’Dwyer, 2000) and reptiles (Dorrough & Ash, 1999; Melville et al., 2007), many of which are of high conservation concern. For example, a phylogenetic study of VIC and ACT/NSW populations of the grassland earless dragon (*T. pinguicolla*) found major genetic structure between the regions, dating back to the late Pliocene (Melville et al., 2007).

Large-scale connection and disconnection of grassland habitats across south-eastern Australia, driven by changing climate, were likely drivers of these biogeographical patterns. Periods of forest expansion and contraction and marine incursions in near-coastal part of the species’ range during late Miocene to mid-Pleistocene (0.7–5 Ma) glacial oscillations likely repeatedly caused *R. leptorrhynchoides* distribution to contract to the grassy woodlands of ACT and the grassy plains of VIC, promoting divergence (Bowler, 1982; Markgraf et al., 1995; Jones, 1997). Few isolated patches may have remained outside these strongholds, such as the one recorded in the Gippsland Plains of eastern VIC (Young et al., 1999). Because the vast majority of intervening forest habitat is currently unsuitable for the species, and given the species’ low dispersal ability (Morgan, 1995), gene flow between north and south likely ceased at the last such grassland contraction, possibly during the Holocene (last ~10,000 years). The onset of land conversion and grazing by exotic ungulates in the last ~180 years (Powell, 1969; Weaver, 1996) would have resulted in reduced, isolated populations within the centres of distribution (ACT and the grassy plains of VIC), leading to the strong drift inferred here.

### Isolation and differentiation of populations with genetic drift

Similar to other outcrossing plant species (Nybom & Bartish, 2000), differences among individuals within populations accounted for the majority of variation in *R. leptorrhynchoides*. Our data implicate genetic drift in isolation as the driving force behind this differentiation, particularly within the southern NSW locations of CF, BB and MI. CF was previously identified as isolated, using STRUCTURE analysis (Pickup et al., 2012).

Loss of genetic diversity through drift is particularly detrimental in self-incompatible species, including *R. leptorrhynchoides*, owing to the loss of variation at the *S*-locus, which controls self-incompatibility (Pickup & Young, 2008; Young & Pickup, 2010). If the number of *S*-alleles falls too low for the breeding system to be sufficiently successful, small self-incompatible plant populations will be highly vulnerable to extinction (e.g., DeMauro, 1993; Wagenius et al., 2007), as supported for *R. leptorrhynchoides* through stochastic matrix projection models parameterised by data on growth, survival and reproduction of four life stages measured in multiple years for each population of interest (Young et al., 2000). Thus, small self-incompatible populations with low genetic diversity are highly likely to benefit from re-establishing gene flow (Frankham, 2015; Frankham et al., 2017 p. 128). Glasshouse and common garden experiments with *R. leptorrhynchoides* suggest that the species is a good candidate for genetic rescue, with evidence of increased seed set and heterosis following inter-population mixing, even between northern and southern genetic clusters (Pickup & Young, 2008; Pickup et al., 2013). Re-establishing natural gene flow among isolated populations such as CF, BB and MI may be challenging in the human impacted landscapes that drove their isolation and population contractions, given the species biology. However, at least in the short term, genetic restoration could be achieved relatively easily by in situ plantings.

### High genetic diversity retained in small Victorian populations

Plant species with declining population sizes are expected to have lower genetic diversity due to drift within populations, compared to commoner and more-widespread species (Frankham, 1996; Cole, 2003). However, our data showed no correlation between estimated population size and genetic diversity (Fig. 1 and Supplementary Fig. S7). Despite their small and declining size, we found that *R. leptorrhynchoides* in VIC have so far retained high levels of genetic diversity relative to their population size, similar to the findings based on allozymes and microsatellites (Young et al., 1999; Pickup et al., 2012). In our data, TR (population size 591 plants) and SA (18) have the highest levels of heterozygosity and allelic richness, higher than those in populations of comparable size in ACT/NSW such as CF (306) and MI (13) as well as those of much larger size such as GU (~90,000) and MJ (~28,000). High genetic diversity and low differentiation in small VIC populations could reflect population reductions being too recent for loss of genetic diversity through drift (Young et al., 1999; Chen et al., 2017). TR and SA are located on the fringes of Melbourne city, are the last known survivors among populations lost since 1950 (Bull & Stolfo, 2014) and have been subject to very recent decline (recent TR population size is ~60% of its size of >1000 in 2002 and SA is ~13% of the 137 reproductive individuals in 2000, Brown & Young, 2000). Together with this relatively recent bottleneck due to human habitat destruction, diversity is likely buffered by the relatively long generation time of *R. leptorrhynchoides* (7–15 years in the wild) compared to the duration of habitat destruction (regionally <200 years; locally decades), and relatively long longevity (>10 years; Scarlett & Parsons, 1990), as seen in other plant species (Lippé et al., 2006; Aægisdóttir et al., 2009). Self-incompatibility tends to act to retain genetic variation in populations by promoting outbreeding, which equalises family sizes, reduces genetic drift and promotes natural selection (Delph & Kelly, 2014). The negative frequency-dependent selection acting on *S*-alleles buffers rare alleles from loss and can act to maintain high diversity at nearby genomic regions (Glémin et al., 2005; Charlesworth, 2006). Although these processes will occur in all natural populations, they may have slowed loss of diversity of only very recently bottlenecked VIC populations but not some ACT/NSW populations that have been small—and thus experienced strong drift and weak selection—for a longer time.

Presently, *R. leptorrhynchoides* is of high conservation concern in VIC, where recruitment is low. Given the ongoing decline and fragmentation of these populations, coupled with the limited dispersal ability of this species, it is likely that without intervention these populations will become differentiated through loss of diversity due to genetic drift as suggested by our data for some populations in the northern region.

## Conclusions and conservation implications

Many northern (ACT/NSW) populations of *R. leptorrhynchoides* are declining and losing genetic diversity via drift, with measurable negative fitness effects (present data; Pickup & Young, 2008). For example, isolation and low genetic variation of the small NSW populations of BB, MI and CF indicate that without intervention they are vulnerable to extinction. While such inbred populations are expected to gain fitness through gene flow from genetically diverse, differentiated populations of their region, in the absence of outbreeding depression, genetic rescue effects are predicted to be even greater using source populations that are more genetically variable and diverged from the recipients (Frankham, 2015; Frankham et al., 2017). Benefits can be substantial even from differently adapted populations (Kronenberger et al., 2017). Consistent with these expectations, breeding experiments in *R. leptorrhynchoides* showed only positive fitness consequences of crossing between northern and southern populations, and greater fitness gain when the source population had higher genetic diversity and lower inbreeding (Pickup & Young, 2008; Pickup et al., 2012, 2013). Thus, the high levels of genetic diversity and unique variation in the small, isolated VIC populations of *R. leptorrhynchoides* highlight their potential value as a key resource for active genetic management of northern populations of this endangered species suffering genetic erosion. Ex situ conservation activities are ongoing for these populations and should remain a priority.

Due to their small and declining population sizes, VIC populations are vulnerable to extinction through environmental stochasticity, and would benefit from demographic rescue. The surprisingly high genetic diversity at TR and SA given their census sizes indicate that demographic augmentation may be of greater urgency than is elevating genetic diversity. Nonetheless, both locations will be losing genetic variation by drift, and SA in particular comprises so few individuals that low *S*-allele diversity may be limiting reproduction (Young et al., 2000). Both VIC populations are sufficiently small to hinder adaptation to new environments, and because they are genetically similar to each other, genetic augmentation from the warmer and drier north could also be considered as a means to help counter changing climate and weather (Frankham et al., 2017). Such decisions can be made by weighing up the risks of genetic erosion from not mixing gene pools versus any possible genetic harm from mixing them (Ralls et al., 2018; Liddell et al., 2020).

## Supplementary information

Rodger et al. HDY-20-A0167RRR Supplementary Material

## Data Availability

DArT genotypes and associated geographic data for each individual are available at Bridges Monash University research repository 10.26180/5ea1603edd3a1. Raw DArT read data are at 10.26180/5ce65bf202a3e.
