# Supplementary material for: Evolutionary history and genetic connectivity across highly fragmented populations of an endangered daisy
Source: Heredity (Edinb). 2021 Feb 19;126(5):846–58. doi: 10.1038/s41437-021-00413-0 (PMC8102499; doi:10.1038/s41437-021-00413-0)
Supplement: Supplementary file 1 — Rodger et al. HDY-20-A0167RRR Supplementary Material [file 41437_2021_413_MOESM1_ESM.docx]

Evolutionary history and genetic connectivity across highly fragmented populations of an endangered grassland daisy – Rodger *et al.*

**Supplementary Information**

**Methods S1: SNP development**

DArTseq uses complexity-reduction methods with combinations of restriction enzymes that target low-copy genomic regions to detect large numbers of informative SNPs within 69 bp sequences (Kilian et al. 2012). Four complexity-reduction methods comprising different combinations of restriction enzymes were tested to optimise representation and genome coverage. The restriction enzyme combination *Pst*I-*Mse*I was applied for genotyping. DNA samples were processed in digestion/ligation reactions as detailed previously (Kilian et al. 2012). Fragments were amplified in 30 rounds of PCR with an initial 94⁰C denaturation step for 1 min, 30 denaturation steps at 94⁰C lasting 20 s, 30 s annealing at 58⁰C, 45 s extension at 72⁰C, and a final extension step at 72⁰C. After PCR, equimolar amounts of PCR products were pooled and applied to an Illumina c-bot for bridge PCR for sequencing on an Illumina Hiseq 2500. Single-read sequences were generated with 77 cycles.

The resulting sequences were filtered using the DArT proprietary primary pipeline to remove poor quality sequences and apply more stringent filtering criteria to the barcode region compared to the rest of the sequence (minimum barcode Phred score 30, pass percentage 75; minimum whole-read Phred score 10, pass percentage 50). This higher stringency allowed for reliable assignment of sequences to the same sample. Identical sequences were collapsed into tags, which served as templates against which low quality bases in singleton tags were corrected. SNP calling was performed using the DArT proprietary pipeline, which clusters sequences then parses clusters into separate SNP loci using a range of technical parameters, balancing read counts per allelic pair. Scoring consistency of alleles (repeatability) was determined using technical replicates for 25% of samples.

**Supplementary figures and tables**


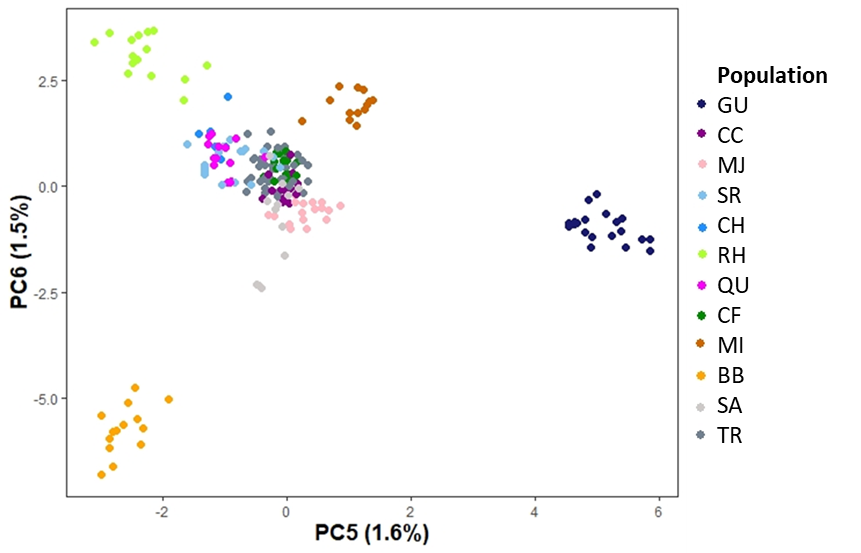


Figure S1. Principal coordinate analysis (PCoA) axes 5 and 6, derived from 12,965 SNPs for all 12 sampled populations. Population codes correspond to those presented in Table 1.


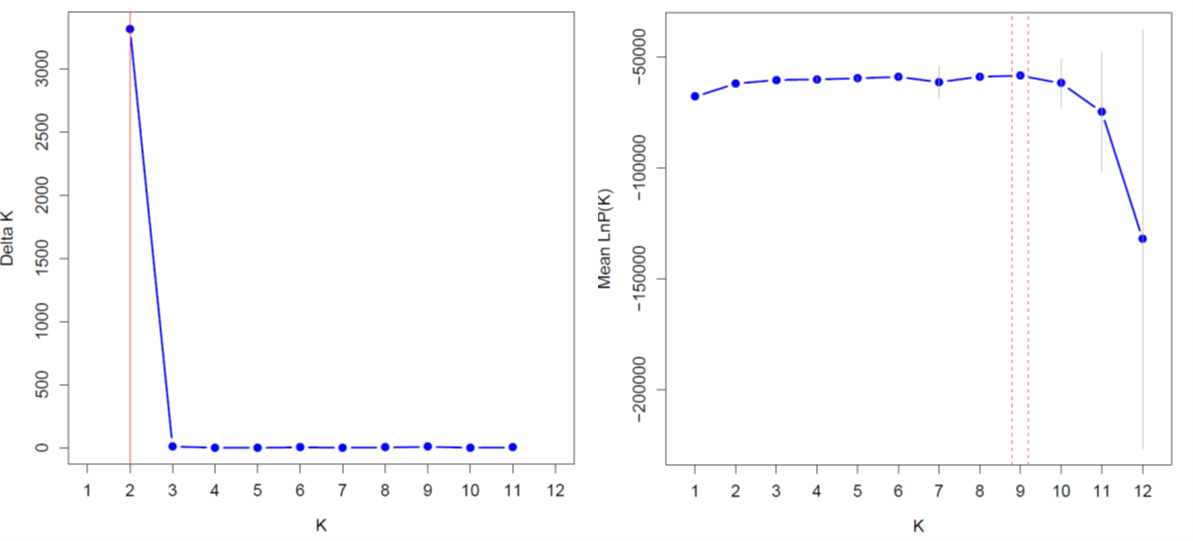


Figure S2. Optimal number of genetic clusters by the ln Pr(X|K) method (left) and Delta K method (right) for the STRUCTURE analysis of all 12 populations for 1,889 SNPs with no missing data. Plots produced by StructureSelector.

Figure S3. Results of the STRUCTURE analysis up to K=6 for all 12 populations of *R. leptorrhynchoides* for 1,889 SNPs with no missing data. Plots produced by CLUMPAK.


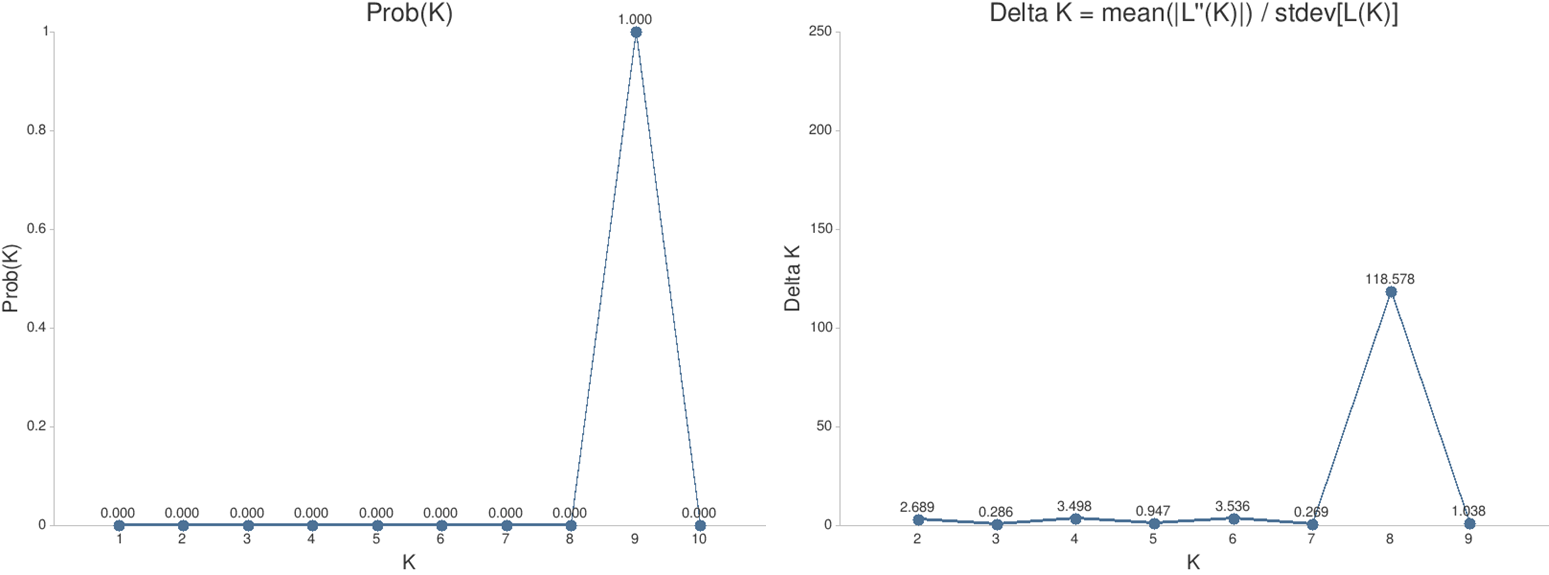


Figure S4. Optimal number of genetic clusters by the ln Pr(X|K) method (left) and Delta K method (right) for the STRUCTURE analysis for 10 ACT/NSW populations genotyped for 1,487 SNPs with no missing data (fewer than the 1,889 loci used for the 12-population analysis, owing to different polymorphisms). Plots produced by CLUMPAK.


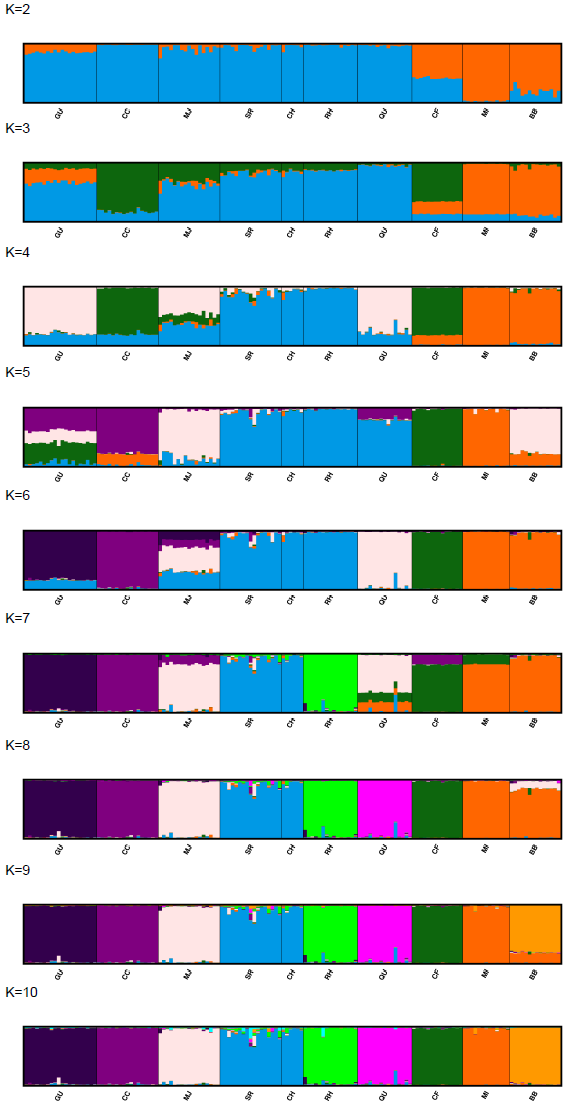


Figure S5. Results of the STRUCTURE analysis up for 10 ACT/NSW populations of *R. leptorrhynchoides* for 1,487 SNPs with no missing data (fewer than the 1,889 loci used for the 12-population analysis, owing to different polymorphisms). Plots produced by CLUMPAK.


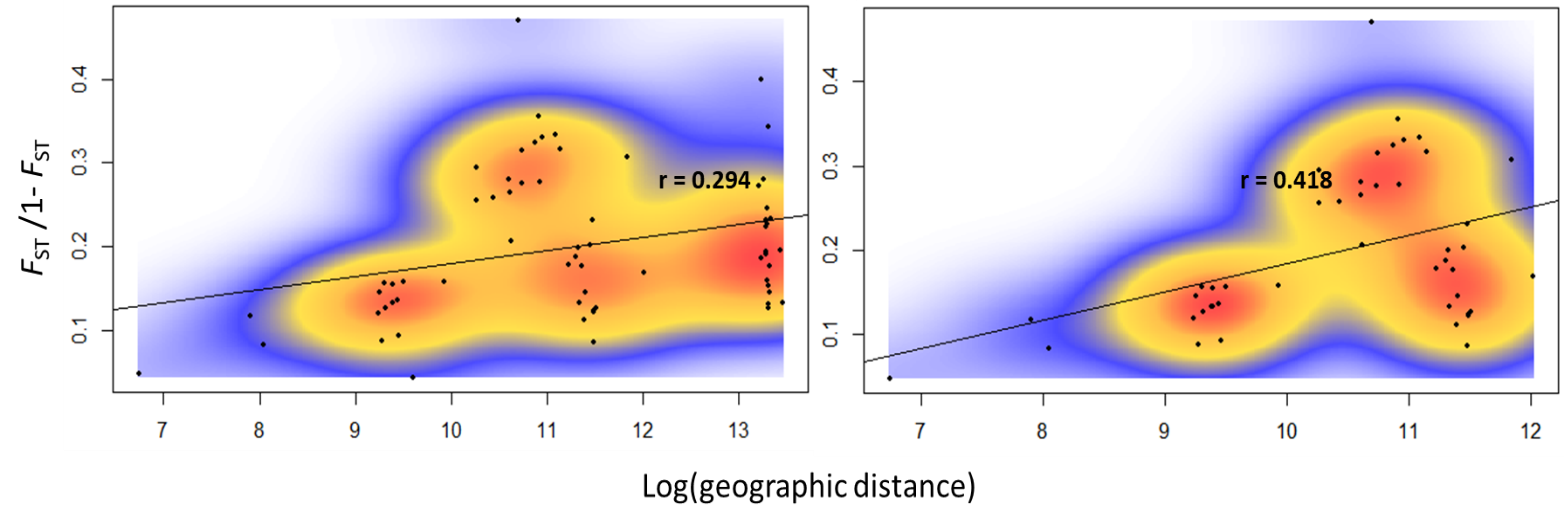


Figure S6. (a) Isolation by distance plot for 12 *R. leptorrhynchoides* populations using 12,965 loci (p = 0.109) and (b) for 10 ACT/NSW populations using 8,232 loci (p = 0.057).

Figure S7. Estimated population size of 12 *R. leptorrhynchoides* populations compared to their observed heterozygosity (*H*_o,_ black dots).


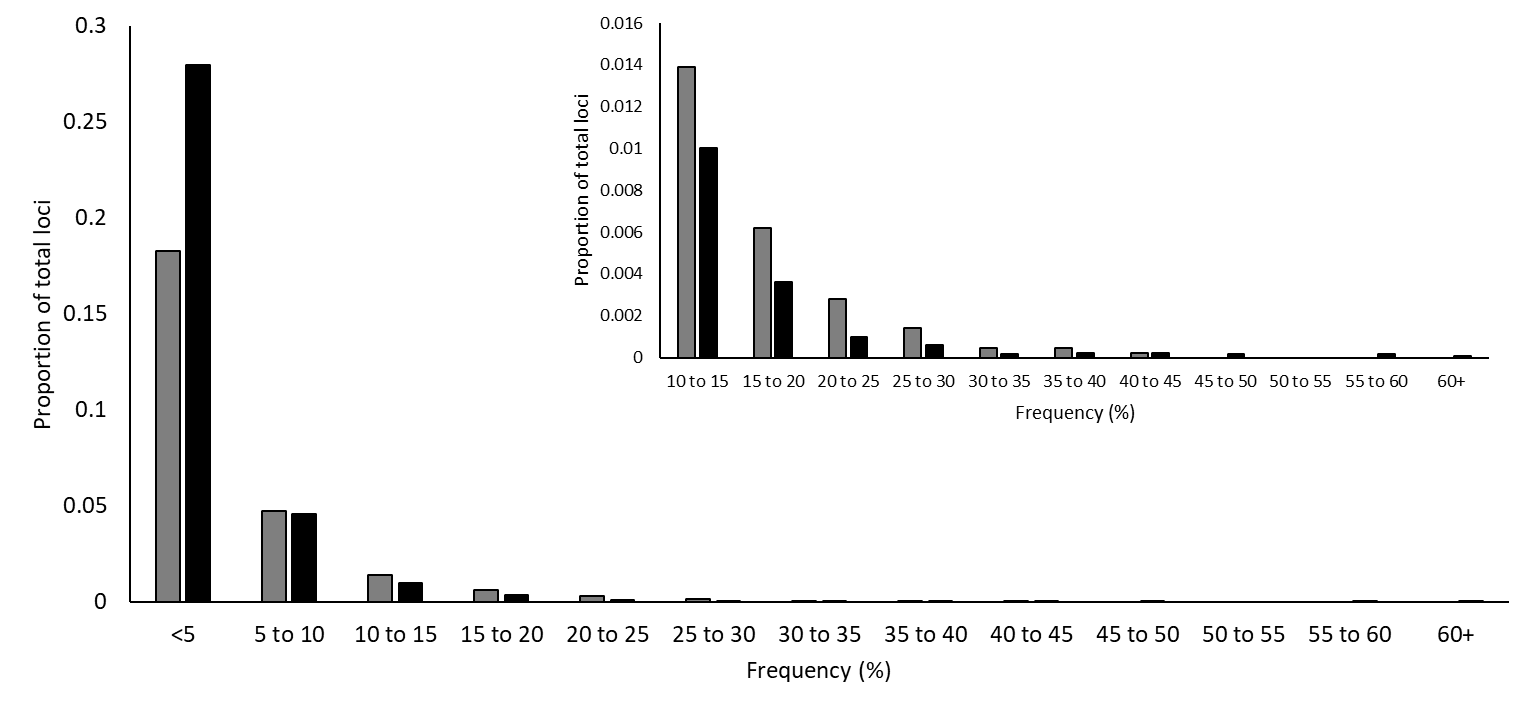


Figure S8. Frequencies of alleles unique to VIC (grey bars), ACT/NSW (black bars) and their proportion of the total loci present (12,965), excluding loci with only one individual carrying a private allele (since unique singletons can only be private). Inset figure shows the proportion of total loci with unique alleles with frequencies above 10% in the sample so that the right-hand tail is more visible.


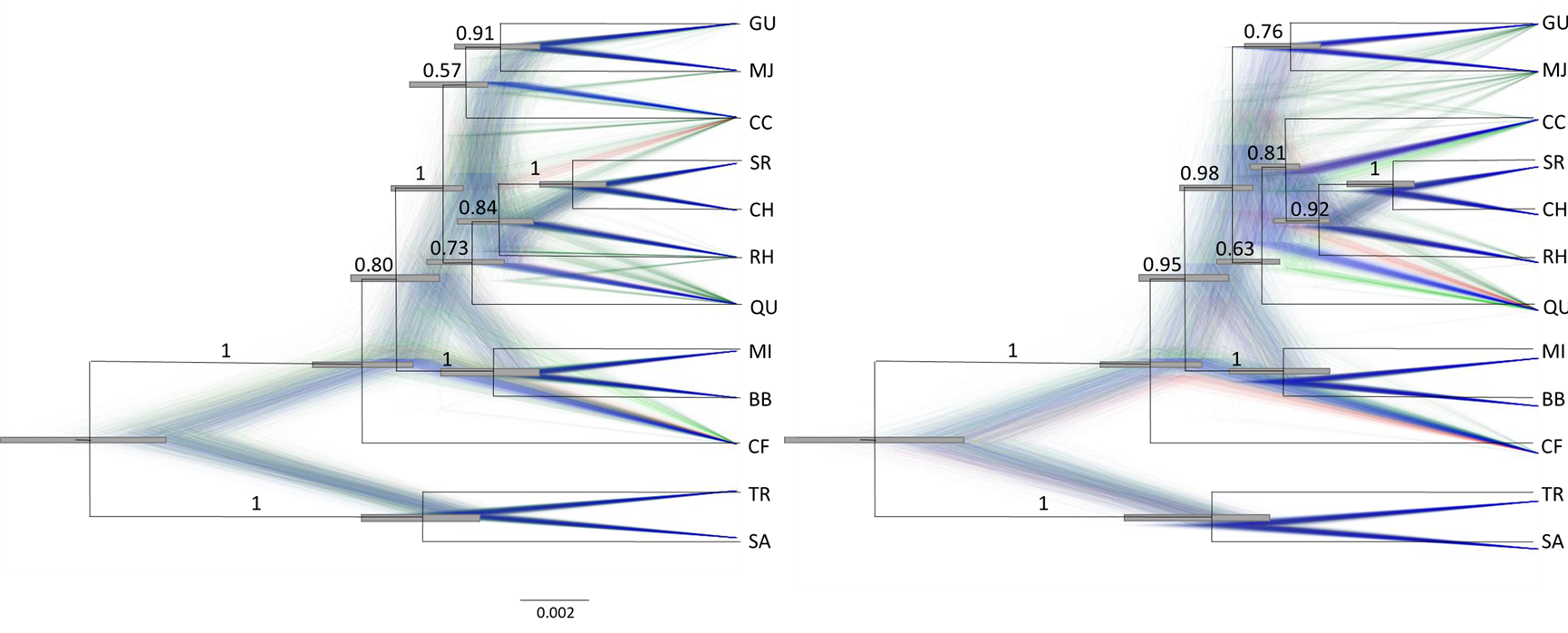


Figure S9. Alternative combined SNAPP population trees of *R. leptorrhynchoides* sampling locations based on four different random individuals and 2,000 different random SNPs. The tree on the left is presented in the main MS as Fig. 4. Maximum clade-credibility tree generated in FigTree v. 1.4.4 (https://github.com/rambaut/figtree/releases) is in black with 95% HPD for height at each node indicated by the grey bar. Branch lengths are measured in expected substitutions per site. Densitree of superimposed gene trees recorded during the MCMC analysis visualises the range of alternative topologies, indicative of past gene flow. Gene trees shown in blue lines indicate most frequent trees, next most frequent are red, third most are green. Population codes correspond to those presented in Table 1.


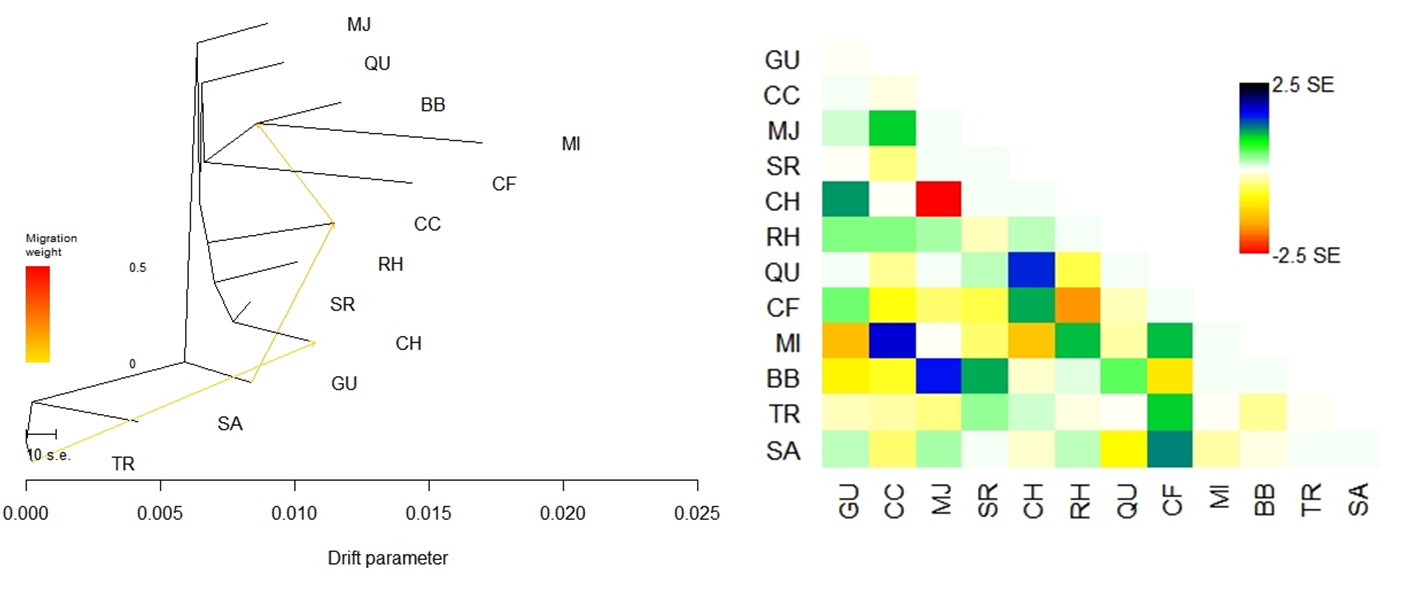


Figure S10. TreeMix analysis of *R. leptorrhynchoides* based on 12,965 SNPs showing the unrooted maximum likelihood tree with three migration events (indicated by yellow arrows), and the residual fit from the tree. The drift parameter is shown on the x-axis and the scale bar shows 10 times the average standard error of the entries in the sample covariance matrix. On the heatmap, large, positive residuals (blue-black colours) indicate population pairs that are more closely related to each other in the data than suggested by the best-fit tree, and may be candidates for admixture events.

**fastSTRUCTURE analysis**

We confirmed that the use of the no-missing dataset in STRUCTURE was justified by running the full 12,965 loci dataset in fastSTRUCTURE (Raj *et al.* 2014) and compared the results. We conducted ten independent replicates of K = 1 to 12 and used STRUCTURESELECTOR (Li and Liu, 2018) to visualise the output and assess the most likely K by comparing the model complexity that maximized marginal likelihood across replicate runs ("chooseK", Raj *et al.* 2014), as well as with the Delta K method (Evanno *et al.* 2005). We also conducted a hierarchical analysis by investigating clustering within ACT/NSW populations only (K =1 to 10 for 10 populations with 8,720 SNPs) and within VIC populations only (K = 1 to 4 for two populations with 7,808 SNPs).

Similar results were achieved to when using the reduced dataset with no missing data in STRUCTURE but with lower resolution in fastSTRUCTURE; ACT/NSW and VIC populations clustered separately at K = 2. For the analysis restricted to ACT/NSW populations, the likelihood of K (LnPr(X|K)) supported K = 1, and DeltaK method supported K=3 (Supplementary Fig. S11). For K=3, one genetic cluster predominated in CF+GU, another in MI+BB, and the third in the remaining populations (Supplementary Fig. S12). Clustering for K=4 further showed CC as a distinct cluster, but higher K did not show geographically coherent clusters. For the analysis restricted to VIC populations, likelihood of K (LnPr(X|K)) and chooseK methods supported K = 1 (Supplementary Fig. S13).

This analysis did not help resolve geographic population structure as well as the PCoA did.


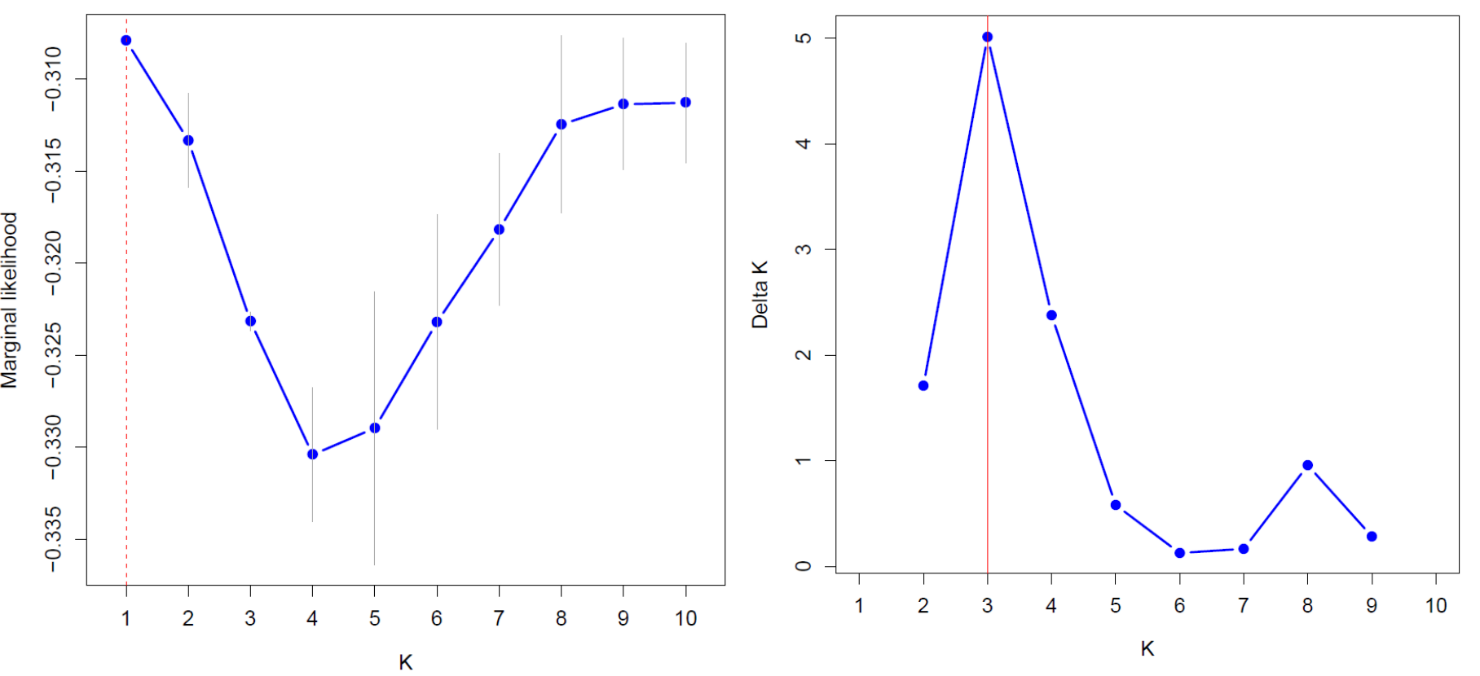


Figure S11. Optimal number of genetic clusters by the ln Pr(X|K) method (left) and Delta K method (right) for the fastSTRUCTURE analysis for 10 ACT/NSW populations with 8,270 SNPs. Plots produced by StructureSelector.


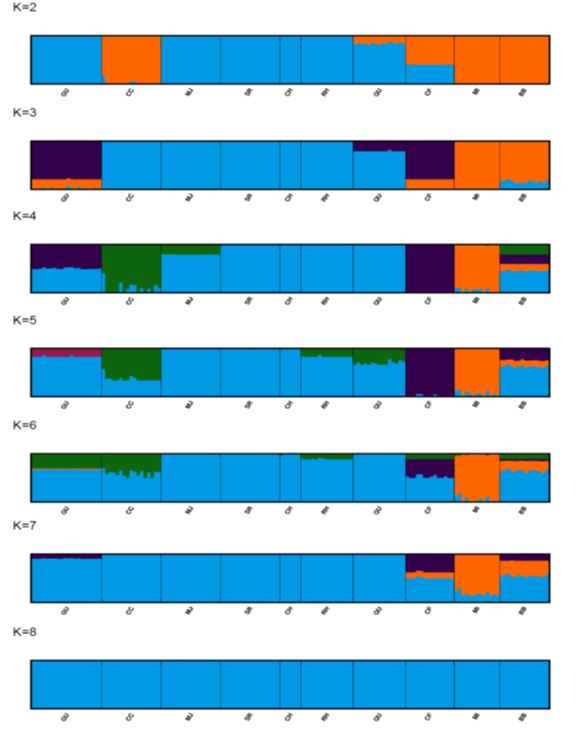


Figure S12. fastSTRUCTURE output plots for *R. leptorrhynchoides* populations based on 8,720 SNP dataset for 10 ACT/NSW populations with an optimum *K* value of 1, according to the chooseK method.


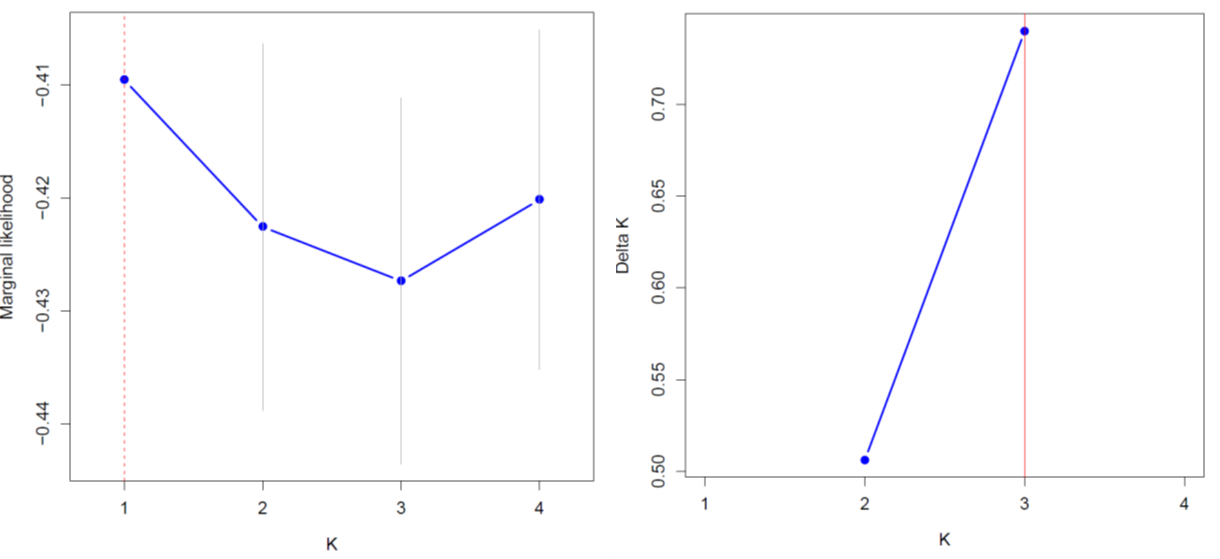


Figure S13. Optimal number of genetic clusters by the ln Pr(X|K) method (left) and Delta K method (right) for the fastSTRUCTURE analysis for two VIC populations with 7,808 SNPs. Plots produced by StructureSelector.


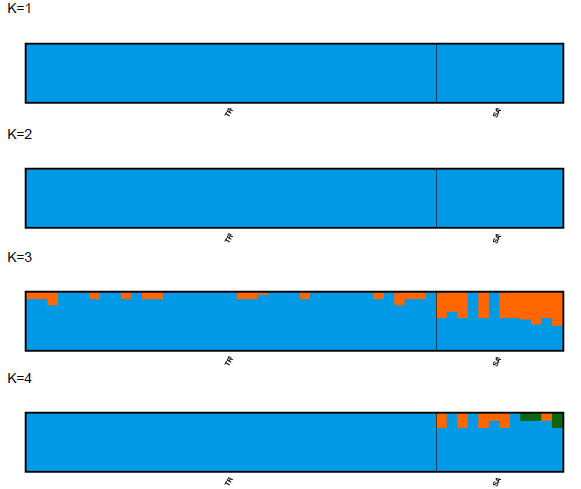
Figure S14. fastSTRUCTURE output plots for two VIC *R. leptorrhynchoides* populations based on 7,808 SNP dataset with an optimum *K* value of 1, according to the chooseK method.

**No-missing data dataset analyses**


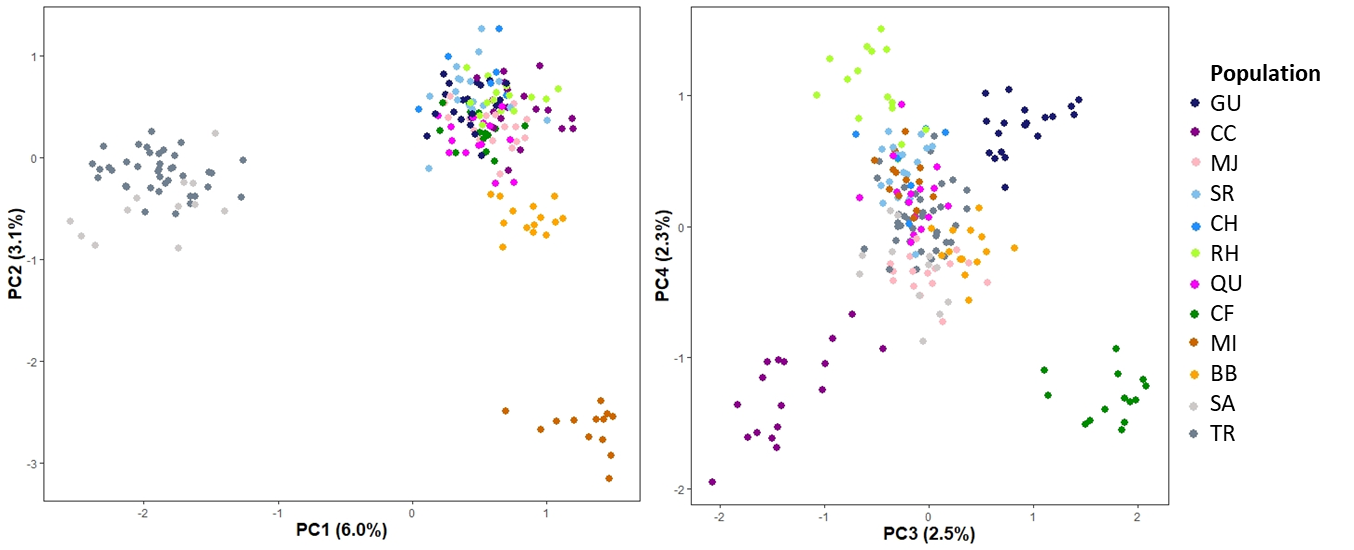


Figure S15. Principal coordinate analysis (PCoA) derived from 1,889 SNPs with no missing data for all 12 sampled populations.


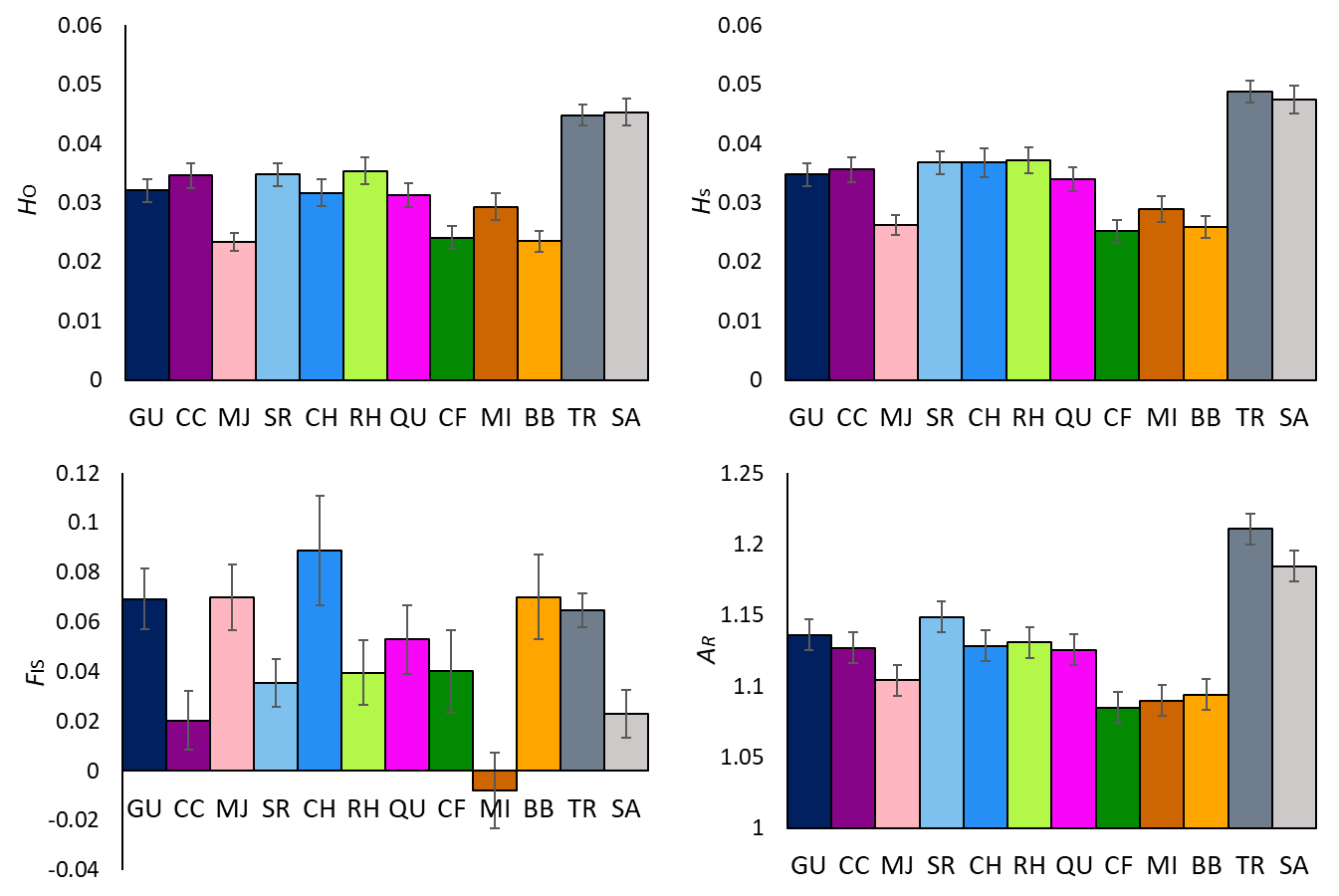


Figure S16. Summary of genetic diversity estimates for *R. leptorrhynchoides* populations derived from 1,889 SNPs with no missing data. *H*_o_ – observed heterozygosity; *H*_S_ – gene diversity, *A_R_* – allelic richness.

Table S2. Pairwise Jost’s D values among sampled populations of *R. leptorrhynchoides* based on 12,965 SNPs. Bold values are significant (p < 0.05).


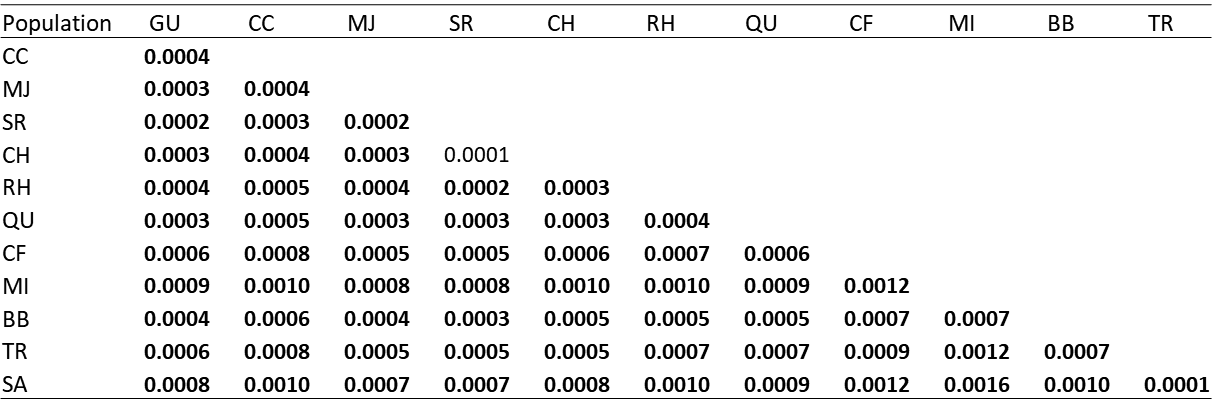


Figure S17. Correlation between pairwise *F*_ST_ and Jost’s D for 12 *R. leptorrhynchoides* populations using 12,965 SNPs.

Figure S18. Correlation of *H*_o_ in black (p = 0.003) and *A*_R_ in grey (p < 0.001) against historical effective population size θ from SNAPP.

Table S3. Genetic diversity estimates for 12 *R. leptorrhynchoides* populations using the full 12,965 SNP dataset and the 1,889 SNP dataset with no missing loci, as well as θ estimates from SNAPP (effective population size scaled by mutation rate).

|  | Full dataset | | | | No missing data set | | | | θ |
| --- | --- | --- | --- | --- | --- | --- | --- | --- | --- |
| Population | *H*O | *HS* | FIS | AR | *H*O | *HS* | FIS | AR |  |
| GU | 0.029 | 0.048 | 0.306 | 1.048 | 0.032 | 0.035 | 0.069 | 1.136 | 0.068 |
| CC | 0.031 | 0.048 | 0.261 | 1.047 | 0.035 | 0.036 | 0.02 | 1.127 | 0.063 |
| MJ | 0.021 | 0.04 | 0.358 | 1.039 | 0.023 | 0.026 | 0.07 | 1.104 | 0.066 |
| SR | 0.03 | 0.049 | 0.29 | 1.048 | 0.035 | 0.037 | 0.035 | 1.149 | 0.068 |
| CH | 0.029 | 0.047 | 0.277 | 1.044 | 0.032 | 0.037 | 0.089 | 1.129 | 0.064 |
| RH | 0.03 | 0.047 | 0.278 | 1.046 | 0.035 | 0.037 | 0.04 | 1.131 | 0.066 |
| QU | 0.027 | 0.046 | 0.318 | 1.045 | 0.031 | 0.034 | 0.053 | 1.126 | 0.065 |
| CF | 0.023 | 0.036 | 0.297 | 1.035 | 0.024 | 0.025 | 0.04 | 1.085 | 0.049 |
| MI | 0.027 | 0.038 | 0.221 | 1.037 | 0.029 | 0.029 | -0.008 | 1.09 | 0.046 |
| BB | 0.022 | 0.04 | 0.354 | 1.039 | 0.024 | 0.026 | 0.07 | 1.094 | 0.054 |
| TR | 0.038 | 0.064 | 0.302 | 1.064 | 0.045 | 0.049 | 0.065 | 1.211 | 0.087 |
| SA | 0.035 | 0.061 | 0.309 | 1.059 | 0.045 | 0.047 | 0.023 | 1.185 | 0.083 |

**References**

Evanno G, Regnaut S, Goudet J (2005) Detecting the number of clusters of individuals using the software STRUCTURE: A simulation study. *Mol Ecol* **14**: 2611–2620

Li Y, Liu J (2018) STRUCTURESELECTOR: A web-based software to select and visualize the optimal number of clusters using multiple methods. *Mol Ecol Resour* **18**: 176–177

Raj A, Stephens M, Pritchard JK (2014) fastSTRUCTURE : Variational Inference of Population Structure in Large SNP Data Sets. *Genetics* **197**: 573–589
